# Use of the ‘patient journey’ model in the internet-based pre-fitting counseling of a person with hearing disability: lessons from a failed clinical trial

**DOI:** 10.1186/1472-6815-14-3

**Published:** 2014-04-07

**Authors:** Vinaya Manchaiah, Jerker Rönnberg, Gerhard Andersson, Thomas Lunner

**Affiliations:** 1 Department of Vision and Hearing Sciences, Anglia Ruskin University, Cambridge, UK; 2 Linnaeus Centre HEAD, Swedish Institute for Disability Research, Department of Behavioural Sciences and Learning, Linköping University, Linköping, Sweden; 3 Department of Clinical Neuroscience, Division of Psychiatry, Karolinska Institutet, Stockholm, Sweden; 4 Eriksholm Research Centre, Oticon A/S, 20 Rørtangvej, Snekkersten, Denmark

**Keywords:** Hearing disability, Patient journey, Pre-fitting counseling, Failed clinical trial, Dropouts, Treatment compliancy

## Abstract

**Background:**

Persons with a hearing impairment have various experiences during their ‘journey’ through hearing loss. In our previous studies we have developed ‘patient journey’ models of person with hearing impairment and their communication partners (CPs). The study was aimed to evaluate the effectiveness of using the patient journey model in the internet-based pre-fitting counseling of a person with hearing disability (ClinicalTrials.gov Protocol Registration System: NCT01611129, registered 2012 May 14).

**Method:**

The study employed a randomized controlled trial (RCT) with waiting list control (WLC) design. Even though we had intended to recruit 158 participants, we only managed to recruit 80 participants who were assigned to one of two groups: (1) Intervention group; and (2) WLC. Participants from both groups completed a 30 day internet-based counseling program (group 2 waited for a month before intervention) based on the ‘patient journey’ model. Various outcome measures which focus on self-reported hearing disability, self-reported depression and anxiety, readiness to change and self-reported hearing disability acceptance were administered pre- and post-intervention.

**Results:**

The trial results suggest that the intervention was not feasible. Treatment compliancy was one of the main problems with a high number of dropouts. Only 18 participants completed both pre- and post-intervention outcome measures. Their results were included in the analysis. Results suggest no statistically significant differences among groups over time in all four measures.

**Conclusions:**

Due to the limited sample size, no concrete conclusions can be drawn about the hypotheses from the current study. Furthermore, possible reasons for failure of this trial and directions for future research are discussed.

## Background

Hearing impairment is one of the most common chronic conditions in adults and can have physical, mental and social consequences for both persons with hearing impairment and their communication partners (CPs). Despite these consequences only a small percentage of those with hearing impairment tend to seek help and uptake audiological interventions [1]. Various factors may influence the help-seeking behavior of persons with a hearing impairment, with self-reported hearing disability being the most important [2]. Self-reported hearing disability is reported to be more common than the hearing impairment as measured by audiometric testing [3]. Furthermore, it is suggested that audiological rehabilitation should be based on perceived difficulties rather than the severity of hearing impairment [4].

There are a range of audiological interventions focusing on different aspects of hearing impairment, such as hearing status, psychosocial needs and supporting enablement at workplace. Even though there is information provision to persons with a hearing impairment before and after the hearing tests, generally most counseling programs are post hearing aid fitting with an emphasis on both technological and psychosocial aspects. The focus of pre-fitting counseling program is to support the person with hearing disability in terms of social and emotional needs, modify attitudes and motivation, and to provide information about choice of interventions [5]. A recent study suggested that older adults like to have more information both before and after the hearing aid fitting [6]. However, studies on pre-fitting counseling programs are rather limited [7-11]. Furthermore, even though most of such counseling programs are offered face-to-face in clinical situations, there seems to be a recent trend to offer such programs using internet or telephone [12-15]. This may bring advantages in terms of cost-effectiveness and also flexibility in terms of participation.

Person with hearing impairment may have various experiences during their ‘journey’ through hearing loss. The experiences patients go through during a disease/acquired health condition and treatment are referred to as ‘patient journey’. It has become common in recent years to study the patient journey of various chronic conditions [16-23]. In our previous studies we have developed patient journey models of person with hearing impairment and their CPs [24-27]. It is suggested that these models could be helpful to train hearing healthcare professionals and also as a counseling tool to be used with patients and their family members [24-27]. Moreover, the patient journey model could be a useful tool for pre-fitting counseling of person with hearing disability [28]. Furthermore, considering the significant number of those in this population may not have consulted hearing healthcare professionals and unlikely to have had their hearing assessed, we have to rely on self-reported hearing disability.

This paper presents the results of a randomized controlled trial (RCT) which intended to evaluate the effectiveness of using the ‘patient’ journey model in the internet-based pre-fitting counseling for person with hearing disability [28]. In addition, possible reasons for failure of this trial and directions for future research are discussed.

## Method

### Study design and participants

**Figure 1:**
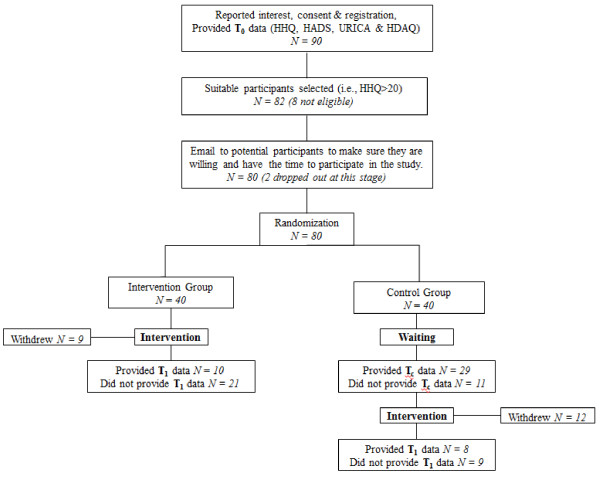
Flow chart showing the study design.

The study employed a RCT design with Waiting List Control (WLC). Figure 1 shows the flow chart of the study design. Detailed information about the study design and the protocol can be found in Manchaiah et al. [28]. Ethical clearance was obtained from the Research Ethics Committee, College of Human and Health Sciences, Swansea University. Study advertisement was made through various sources including national newspaper, hearing loss charity websites (i.e., Action on Hearing Loss and Hearing Link) and also through local GP practice notice boards. Interested participants who were noticing hearing difficulties, not using hearing aids and also had access to internet were encouraged to access the study website using the URL supplied. Website front page provided detailed information about the study and those who decided to take part were requested to provide consent and also complete few questionnaires online.

The participants *inclusion criteria* included: age over 18 years; noticing symptoms of hearing disability; access to internet. Also, the e*xclusion criteria* included: already using hearing aids; HHQ scores 20 or below; and those with additional disabilities (e.g., visual impairment, learning disability, dementia, and so on) which may affect individuals’ ability to participate in an internet-based program. We had initially planned to recruit 158 participants based on the sample size calculation [28]. However, it proved difficult to recruit sufficient number of participants even with repeated advertisements. A total of 90 participants completed the initial screening questionnaire out of whom 82 were eligible. However, we emailed them to check if they had the time to participate in the study and at that stage, 2 participants dropped out. The remaining 80 participants were randomly assigned to one of the two groups: (1) Intervention group; and (2) WLC. Randomization was done using computer-generated random numbers and researcher-blinded. Participants from intervention group completed the 30 days (4 weeks) counseling program while the WLC group waited for 30 days without any active treatment. However, participants in WLC were advised to read generally about hearing loss and its treatment during the waiting period. Furthermore, the participants in WLC group were also given the counseling program after the waiting period. Participants from both groups completed questionnaires pre and post-counseling.

Due to the nature of the study, it was not possible to blind the participants and the clinician. However, unique reference numbers were given to each participant throughout the study which ensured anonymity to some degree. As the emails of participants (which may have their name) were stored in the system it was not completely anonymous. Considering that the participants did not have any direct contact with the researcher there was no control over co-intervention. However, participants were requested to keep the clinician informed if they were to engage in any other activities which may have influenced the outcome of this study (e.g., using hearing aids, taking part in other counseling or education programs). In addition, regular emails were sent to participants to check their progress and also to provide any information necessary.

### Intervention

The intervention involved a pre-fitting counseling program delivered online using the internet-based counseling protocol system which was developed in Sweden [29]. Following the online registration and completion of assessment battery, participants deemed eligible were given a password and an unidentifiable username (e.g., 1037) which gave them access to the treatment program and platform to communicate with the therapist. However, the email and other details were stored in the online system. Technical support was available if the participants encountered technical difficulties.

The counseling program was expected to be completed within 30 days which was developed based on the previous studies on patient journey notions [24-27] and also considering principles of health behavior change models [30]. Generally, the main focus of this program was on the lived experiences of the person with hearing disability with significant emphasis on ‘self-reflection’ rather than technical information such as audiogram and hearing aids. The program involved four stages with designated internet sessions and additional tasks which the participants could complete in their own time. The sessions include: Stage 1 – introduction to the concept of the ‘patient journey’ and presenting to the participants a series of questions which may help them to explore their ‘journey’ through hearing loss and complete the task by reporting their journey through online system; Stage 2 – the ‘patient journey’ model of person with hearing impairment and two case examples were presented. The person with hearing disability were advised to compare their ‘journey’ (i.e., previously reported in the online system) to the model presented and identify the similarities and differences; Stage 3 – the ‘communication partners’ journey’ model was presented and the person with hearing disability were asked to consider how interactions between him/her and the CP may affect various things in the physical, mental and social domains; and Stage 4 – participants were encouraged to think about how the person with hearing disability and CP may influence each other during their ‘journey’ through hearing loss, how they can overcome some of the difficulties they may be experiencing and to think about the potential benefits and the challenges from the audiological management.

Materials at each stage were supplemented with a short video which briefly explain the tasks. The information presented in the video was also available in text. Throughout this process, the participants were advised to reflect (a simple guide for reflection was provided) and maintain notes. This reflection exercise is to explore the activity limitations and participation restrictions they have and also to explore the ways in which they can overcome them. Participants were able to communicate with the researcher as and when they need using the contact handling system through emails to update their progress with the intervention and seek further assistance. This was encouraged (i.e., at least once a week after completing the task) and offered to all those who had registered as this was possible only by logging into the online system. Those who did not login and dropped out of the study did not have this facility. Furthermore, we had planned to provide the counseling materials only through the internet-based counseling protocol system. We noticed that nearly half of the participants in each group had not logged into the system half way through the program (i.e., two weeks after). For this reason, counseling materials (pdf versions) were sent to participants’ emails directly using the online system encouraging them to participate in the study. However, this did not make any difference in number of people using the program.

The study protocol was registered in the http://ClinicalTrials.gov Protocol Registration System before the start of the study (Registration number: NCT01611129; Study ID number: FAS-IT-03).

### Outcome measures

Hearing Handicap Questionnaire (HHQ) was the primary outcome measure used and the secondary outcome measures include: Hospital anxiety and Depression Scale (HADS), University of Rhode Island Change assessment (URICA) scale and the Hearing Disability Acceptance Questionnaire (HDAQ).

HHQ is an instrument to measure self-reported hearing disability [31]. HHQ has 12-items scored on a 5-point Likert scale (1 = never, 5 = almost always). Total scores range from 12 to 60 with higher scores indicative of greater disability. HHQ has single factor structure and a good internal consistency, with Cronbach’s alpha of 0.93 [32].

HADS is a measure for self-reported anxiety and depression [33]. The HADS consists of 14-items divided into two subscales (anxiety and depression). Each item is scored from 0 to 3 (0 = not at all, 3 = most of the time) and the scores range from 0 to 42 with higher scores indicative of more self-reported anxiety and depressive symptoms. HADS has good reliability and acceptable sensitivity and specificity [34].

URICA is one of the most commonly used stages-of-change measure [35,36]. The original URICA scale consists of 32-items with four stages (i.e., precontemplation, contemplation, action and maintenance). However, in this study we used 24-item scale with three stages by removing the ‘maintenance’ stage as it was not appropriate for the study sample. This is a generic measure, so the term ‘the problem’ was replaced by ‘the hearing problem’ makes it suitable for the study population. Each item is rated on a 5-point Likert scale (1 = strong disagreement, 5 = strong agreement) and each sub-scale measures specific aspects. Total scores in each subscale can range from 8 to 40. Readiness to change composite was calculated by adding the sums of subscales contemplation and action stages and then subtracting the sum of precontemplation stage scores (contemplation + action–precontemplation). This scale has been found to have good construct, concurrent and predictive validity [37].

HDAQ is a measure of self-reported hearing disability acceptance which has 7-items with two subscales [38]. The subscale ‘activity engagement’ has 4-items and the subscale ‘avoidance and suppression’ has 3-items. Each item is rated on a 7-point Likert scale (1 = never true, 7 = always true) with items in subscale ‘avoidance and suppression’ having reverse scoring structure. Total scores range from 7 to 49 with higher scores indicative of higher hearing disability acceptance. HDAQ has good internal consistency, with Cronbach’s alpha of 0.86 [38].

### Data analysis

Statistical analysis was performed using the software IBM - SPSS Version 19 for Windows. Descriptive statistics were used to examine demographic factors. An alpha level of .05 was determined as significant for all statistical analyses. Analysis of variance (ANOVA) was performed to study the differences in pre- and post-counseling results.

## Results

Table 1 shows the sample characteristics. The sample characteristics in the originally recruited participants and those included in the analysis did not vary much except for the duration of hearing disability which was not statistically significant.

**Table 1:** Sample characteristics

	**Originally recruited**	**Included in the analysis**
**N**	80	18
**Age (yrs; M ± SD)**	62.70 ± 10.64	62.11 ± 4.31
**Gender (% female)**	47.5	61.1
**Years since hearing disability onset (yrs; M ± SD)**	12.33 ± 11.18	14.72 ± 14.5
**Education (%)**		
▪ Compulsory education	12.5	11.1
▪ Secondary education	50.0	38.9
▪ Tertiary education	37.5	50.0
**Consulted hearing healthcare professional (%)**		
▪ Yes	63.8	44.4
▪ No	36.2	55.6
**Computer experience**		
▪ Basic	32.5	33.3
▪ Intermediate	65.0	66.7
▪ Expert	2.5	0
**Self-reported hearing disability (HHQ; M ± SD)**	36.01 ± 8.74	34.33 ± 7.93
▪ HHQ–Emotional	21.22 ± 4.96	21.00 ± 3.79
▪ HHQ–Social	14.76 ± 4.97	13.33 ± 4.78
**Self-reported anxiety and depression (HADS; M ± SD)**	14.78 ± 7.47	14.33 ± 8.35
▪ HADS–Anxiety	7.13 ± 4.34	6.50 ± 4.55
▪ HADS–Depression	7.65 ± 3.87	7.83 ± 4.29
**Change assessment (URICA)**		
▪ Readiness to Change composite (Scores ± SD)	39.56 ± 8.92	38.72 ± 8.81
**Self-reported hearing disability acceptance (HDAQ; M ± SD)**	36.30 ± 7.72	37.61 ± 7.48
▪ HDAQ–Activity engagement	22.54 ± 4.36	23.00 ± 4.27
▪ HDAQ–Avoidance and suppression	13.66 ± 4.45	14.06 ± 4.45

Treatment compliancy was a major problem in the current study and we also noticed high dropout rates. By the end of the study 21 participants (26.25%) dropped out from the study, 41 participants (51.25%) did not complete the questionnaires and only 18 participants (22.5%) completed both pre and post-counseling questionnaires. Our analysis suggested no differences in participant characteristics among those who withdrawn, those who did not complete questionnaires and those who continued to participate in the study till completion. Email was sent out to those who are withdrawn from the study (i.e., 26.25%) asking for a reason and only half of them provided specific reason. The reasons included: health issues (3.7%); found the task difficult and not relevant (3.75%); Obtained hearing aids (2.5%); family emergency (2.5%) and no reason given (13.75%). The participants who dropped out did not complete the post counseling outcome measure. Most of the participants who did not complete the questionnaires (i.e., 51.25% participants) had also not logged into online protocol system even after repeated reminders. However, those who did not complete the questionnaires generally did not take part in the study as it was evident from the online protocol system that they have either never or occasionally accessed information. Even though 18 participants completed the questionnaires pre-and post-counseling, only a half of them completed the weekly tasks and actively participated in the counseling program.

Table 2 shows the pre-and post-counseling mean scores and standard deviation for both groups. There were no significant difference found between the pre-and post-counselling scores for HHQ [*F*(1,16) = 0.6, *p* = 0.4], HADS [*F*(1,16) = 1.6, *p* = 0.2], URICA [*F*(1,16) = 1.2, *p* = 0.3] and HDAQ [*F*(1,16) = 3.0, *p* = 0.1]. There was also no interaction effect seen between groups among these scores.

**Table 2:** Pre and post means and standard deviations of self-reported outcome measures among groups

	**Mean ± SD**				
	**Group 1**		**Group 2**		
	**T**_**0 **_**(pre)**	**T**_**1 **_**(post)**	**T**_**0 **_**(pre)**	**Tc (control)**	**T**_**1 **_**(post)**
**Self-reported hearing disability (HHQ)**	35.80 ± 8.2	34.80 ± 7.7	32.50 ± 7.7	31.50 ± 9.4	32.00 ± 9.4
**Self-reported anxiety and depression (HADS)**	14.50 ± 8.3	16.20 ± 9.1	14.12 ± 8.9	14.13 ± 9.3	14.50 ± 9.5
**Readiness for change (URICA-R)**	40.20 ± 11.1	34.10 ± 14.8	36.88 ± 4.6	42.75 ± 7.0	38.62 ± 10.0
**Self-reported hearing disability acceptance (HDAQ)**	35.50 ± 6.5	34.20 ± 6.1	40.25 ± 8.1	37.63 ± 6.9	38.00 ± 9.1

## Discussion

The study presents the results of a randomized controlled trial which was aimed at evaluating the effectiveness of the ‘patient journey’ model in the internet-based pre-fitting counseling of person with hearing disability. The trial results suggest that the intervention was not feasible. No significant difference was found between treatment and control groups over time. Due to limited sample size no concrete conclusions can be drawn about the hypotheses from the current study. However, the cross sectional data obtained in the beginning of the study (i.e., pre-counseling) could be very useful in better understanding the characteristics of this unique population. Furthermore, there are mixed findings about benefits of pre-fitting counseling programs in the literature and the effects seen seem to be generally small [8-11].

It is not uncommon that the clinical trials fail, but they often do not get reported and this selective reporting has caused bias in published reports [39]. Studies from other health areas have indicated that missing data due to selective dropouts [40,41] and recruitment issues [42] have been the main issues for clinical trials failure. Other common reasons may include: treatment compliancy, funding issues, governance issues, no effect seen and so on. Certainly, there are lessons to be learnt from unsuccessful clinical trials such as this [42-45].

### Possible reasons for failure of this trial

By considering the observations made before, during and after the current trial we propose the following may be some of the possible reasons for the study failure. We can broadly categorize them into issues related to recruitment and retention of participants.

Firstly, due to study design chosen (i.e., internet-based), the researcher did not have any direct contact and/or control over the participants’ behavior. We can assume that in some cases family members of those with hearing disability may have registered online for the study on behalf of the participant even if the person with hearing disability had limited knowledge and skills of internet use. This means the person with hearing disability can only access internet with support of family members. Even though it is not possible to completely avoid this some measures may have helped reduce such instances. For example, telephone calls to potential participants before the start of the counseling program to make sure they are able to use internet and have the time to participate in the study may have helped. In addition, advertising the study in audiology and Ear, Nose and Throat departments and registering them only if they meet the entry criteria may also be a useful approach to adopt. However, it is important to note that internet-based recruitment strategies for studies on hearing impaired population have been successful in few countries including Sweden, Netherlands and Germany [14,46-49].

Secondly, it appears that the internet-usage in older adults in the UK is limited when compared to other developed countries. We noticed that a high number of participants rarely or never logged into online counseling protocol system and also did not respond to the emails sent directly to their inbox. This is also supported by a recent study done in the UK which suggests that the internet use in younger adults (i.e., 50–62 years) and older adults (i.e., 63–74 years) is only 60.9% and 29.8% respectively [50]. Many of them may use the internet occasionally, which may to some degree have been addressed by having the longer duration to counseling program delivery (e.g., 3 months).

Thirdly, counseling materials did not seem to be popular with everyone. This is evident from reports of those participants who withdraw and also from the fact that only half of those who completed the pre- and post-counseling questionnaires actively took part in the counseling program. This may due to the fact that having ‘self-reflection’ as the core concept may not appeal to everyone. Also, many participants may have expected to see some technical details related to hearing loss and hearing aids in the counseling program. This may suggest that it may be appropriate to use information focusing on verities of aspects while developing a counseling program. This issue was considered while developing the counseling materials and the study design. However, decision was made to use only the concept of patient journey to evaluate its effectiveness rather than the effect from composite materials. This issue may pose a dilemma to researchers as to whether they want develop a counseling program which appeals to majority of the population or alternatively find the individuals who may like the aspects of self-reflection and study the effectiveness of the patient journey independently. Furthermore, it is likely that the self-reflection could be unexpected by the persons with suspected hearing problems who are looking for information, rather they may be expecting some action (e.g., treatment recommendations such as hearing aids).

Lastly, the current study population may be considered as a challenging group as they are not actively seeking help due to various factors (e.g., less perceived hearing disability, lack of motivation etc.). The mean age of participants in the study sample was about 62 years. Given that the typical age for first consultation with hearing healthcare professional and also first fitting of hearing aids is approximately 70 to 74 years and with many of these adults experiencing hearing difficulties for an average of 10 years prior to that [37,51], the motivation levels in current study population may have been limited. Alternatively, we can anticipate that only those with higher motivation from the general population may have enrolled in the study and further investigations are necessary to test these assumptions. Further, as the participants in this study may not have necessarily received the diagnosis of hearing impairment they may not have considered they need to go through formal program to resolve their problems.

In summary, despite following the structured approach to conducting clinical trial the current study failed. To some degree this clinical trial had failed even before it got started due to recruitment issues. However, we had anticipated this as one of the potential threats to our study as reported in the study design publication [28].

### Further research

There are limited studies focusing on pre-fitting counseling, so efforts are needed to develop and evaluate such programs as the significant number of those with hearing difficulties does not seek professional help. Further research is necessary to evaluate the use of ‘patient journey’ model in the pre-fitting counseling and also to test the proposed hypotheses. Considering the limited use of internet in some countries especially in older adults it might be appropriate to look into other methods of counseling provision (e.g., face-to-face) and it is worth investigating the user preferences towards treatment mode. Also, as not much is known about the population at early stages of hearing disability who may not seek help actively, research is need to understand their population characteristics and also help-seeking behavior. For this reason, it may be useful to include other measures related to motivation, self-efficacy, etc. which may provide some information about the characteristics of the study population. Also, recruitment of participants using on-line strategies has been criticized by some researchers suggesting that it may not be representative of the general population [52,53] which may support the use of other recruitment options such as primary care settings while conducting future studies, although online recruitment strategies have been found to be effective in other countries [14,46-49].

There are some suggestions made in the literature about front loading the process (i.e., making sure the sequence of actions before the clinical trials start are under control) while conducting clinical trials which may be a useful approach to adopt [54]. Furthermore, it might be worth conducting pilot and/or feasibility study before conducting the RCT [55].

## Conclusions

No concrete conclusions about the hypotheses can be drawn from the current study due to limited sample size. However, the study has helped us identify various issues which may have resulted in the study failure. It appears that evaluating the pre-fitting counseling program is challenging and researchers should take caution while designing clinical trials to avoid some of the common mistakes some of which are reported in this paper.

## Abbreviations

CP: Communication partner; HHQ: Hearing handicap questionnaire; HADS: Hospital anxiety and depression scale; HDAQ: Hearing disability acceptance questionnaire; RCT: Randomized Controlled Trial; URICA: University of Rhode Island Change Assessment Scale; WLC: Waiting list control.

## Competing interests

The authors declare that they have no competing interests.

## Authors’ contributions

VKCM was the main researcher who collected data, analysed results and written up the paper. JR, GA and TL assisted in designing the study, planning the analysis and provided assistance in writing up the manuscript. All authors read and approved the final manuscript.

## Pre-publication history

The pre-publication history for this paper can be accessed here:

http://www.biomedcentral.com/1472-6815/14/3/prepub
